# A Chinese family with Oguchi’s disease due to compound heterozygosity including a novel deletion in the arrestin gene

**Published:** 2012-03-01

**Authors:** Lingli Huang, Wen Li, Weilin Tang, Xiaohua Zhu, Pingbo Ou-yang, Guangxiu Lu

**Affiliations:** 1 Institute of Reproductive and Stem Cell Engineering, Central South University, Changsha, P.R. China; 2 Reproductive and Genetic Hospital of Citic-Xiangya, Changsha, P.R. China; 3 Department of Ophthalmology, the Second Affiliated Xiangya Hospital, Central South University, Changsha, P.R. China

## Abstract

**Purpose:**

Oguchi’s disease is a rare autosomal recessive disease and known to be caused by mutations in the rhodopsin kinase (*GRK1*) gene or the arrestin (*SAG*) gene. *SAG* contains 16 exons and encodes a protein with 405 amino acids. This study was to identify the underlying genetic defects in a non-consanguineous Chinese family with Oguchi’s disease.

**Methods:**

Ophthalmologic examinations including fundus photography and electroretinography (ERG) were performed on all family members. All exons of the *GRK1* gene and the *SAG* gene were amplified with PCR and directly sequenced. Quantitative real-time PCR (qPCR) was performed to screen heterozygous deletions/duplications in the *SAG* gene. Long-range PCR and direct sequencing were further performed to define the breakpoints.

**Results:**

The patient had characteristic clinical features of Oguchi’s disease, including night blindness, normal vision fields, typical fundus appearance with the Mizuo-Nakamura phenomenon, nearly undetectable rod b waves in the scotopic 0.01 ERGs, and nearly “negative” scotopic 3.0 ERGs. No mutations were found in the *GRK1* gene. A heterozygous nonsense Arg193stop (R193X) mutation was found in the *SAG* gene in the patient and the unaffected mother. No pathogenic *SAG* mutations were found in the unaffected father. qPCRs showed a heterozygous deletion encompassing exon 2 of the *SAG* gene in the patient and the unaffected father. Long-range PCR and direct sequencing verified the deletion and revealed the breakpoints of the deletion, skipping a 3,224-bp fragment of the *SAG* gene. The deletion was not detected in 96 unrelated healthy controls. This deletion was predicted to eliminate the exon 2 and the AUG initiate codon in the mature *SAG* mRNA and cause no production of the SAG protein or low-level production of a non-functional truncated protein lacking 134 amino acids in the NH_2_ terminus.

**Conclusions:**

Compound heterozygosity of a nonsense R193X mutation and a heterozygous deletion of 3,224 bp encompassing exon 2 in the *SAG* gene is the cause of Oguchi’s disease in this Chinese family. qPCR analysis should be performed if there is a negative result of the mutation screening of the *SAG* gene in patients with Oguchi’s disease.

## Introduction

Oguchi’s disease (OMIM 258100) is a rare form of stationary night blindness with autosomal recessive inheritance, characterized by a typical clinical feature called the Mizuo-Nakamura phenomenon in which the golden-yellow discoloration of the fundus disappears in the dark-adapted condition and reappears shortly after exposure to light [1]. In addition, patients with Oguchi’s disease usually have night blindness but normal color vision, visual acuity, and visual field [2]. Electroretinographic examinations showed reduced or no rod functions with normal cone functions [2,3].

Two causative genes have been reported for Oguchi’s disease: the arrestin (*SAG*; OMIM 181031) gene [1] and the rhodopsin kinase (*GRK1*; OMIM 180381) gene [4]. Beginning in 1995 with the first report on a homozygous *SAG* frameshift 926delA (formerly referred to as 1147delA) [5-7] mutation in five of six unrelated Japanese patients [1], more *SAG* mutations have been found in Oguchi’s disease, including an additional homozygous frameshift 926delA mutation in seven other Japanese families [8-12], a homozygous nonsense Arg193stop (R193X) mutation in an Indian family [13], a compound heterozygous mutation of a nonsense R175X mutation plus a frameshift 926delA mutation in a Japanese family [14], and a homozygous nonsense R292X mutation in a Japanese family [14] and in a South Asian family [15]. Recently, an additional heterozygous frameshift 926delA mutation without identification of the other mutation in another allele during mutation screening of the *SAG* gene was reported in a Japanese patient [16], suggesting that other types of mutations, undetectable with mutation screening of the *SAG* gene, may exist.

In this study, we report a heterozygous nonsense R193X mutation and a novel heterozygous deletion of 3,224 bp encompassing exon 2 in the *SAG* gene in a Chinese family. The deletion was identified using quantitative real-time PCR (qPCR). To our knowledge, this is the first case found in a Chinese family and is the first report of a novel heterozygous deletion in the *SAG* gene.

## Methods

A Chinese family with a 13-year-old female patient and her unaffected parents participated in this study. This study conformed to the tenets of the Declaration of Helsinki, and the research protocol was approved by the Ethics Committee of the Reproductive and Genetic Hospital of Citic-Xiangya (Changsha, P.R. China). Informed consent was obtained from all family members after an explanation of the purpose of this study was provided.

### Clinical examinations

Family members were clinically examined at the Department of Ophthalmology, the Second Affiliated Xiangya Hospital of Central South University. Ophthalmologic examinations included best-corrected visual acuity (BCVA), slit lamp biomicroscopy, fundus photography, kinetic perimetry, and electroretinography. Kinetic perimetry was performed on a Twinfield perimeter (Oculus Inc., Wetzlar, Germany) using defined stimuli according to the Goldmann standard. Standard full-field electroretinograms (ERGs) were elicited with Ganzfeld stimuli after 30 min of dark adaptation using the commercial ERG system (*RetiPort32*; Roland Consult Systems, Bradenburg, Germany) according to the guidelines of the International Society for Clinical Electrophysiology of Vision (ISCEV)(standard flash, 3 cd·s·m^−2^) [17]. The stimuli were 0.0095 cd·s·m^−2^ for rod stimulation (Scotopic 0.01 ERG) and 3 cd·s·m^−2^ for other standard responses (including scotopic 3.0 ERG, photopic 3.0 ERG, and photopic 3.0 flicker). For photopic ERG, the background luminance was set at 34 cd·m^−2^.

### Mutation screening

All exons of the *GRK1* gene, including exon/intron boundaries, were amplified with PCR and directly sequenced as previously described [18]. All exons of the *SAG* gene with exon/intron boundaries were amplified with PCR using primers (Table 1) designed by Primer 3 and checked by NCBI BLAST for specificity followed by direct sequencing for mutation screening.

**Table 1:** Primers for mutation screening of *SAG*.

**Exon**	**F/R**	**Primer Sequence (5′-3′)**	**PCR size (bp)**
1	F	GCTTGCATAACACCAGGTTCATC	750
	R	CCGCTCACTCCAAGTCTCC	
2	F	TTGTCTTACCTTTCTCCAACCC	255
	R	CCCTCAAAGAGTTTTGATGTTG	
3	F	CATGGATGCCTTAGCTTAGC	217
	R	TAGATTATTAGCAAGGCCAG	
4	F	GGTTTCTTTCATCTTCTCCA	189
	R	CTCTCCTTCCATGTAAATG	
5	F	TTGAAAACCCGTGTTCGCTG	366
	R	TCTATCCCCTTTCCTTTGCC	
6	F	ATATTACTTAATGGAACAGC	213
	R	ACAGAGTAAAACCCTGTTTC	
7	F	CATGTGCCCTGTGTGAGGTG	246
	R	CCACAGAGACAAGGTGGAGG	
8	F	GGAGAGAACAGAAGCCTCCC	300
	R	ATGTAGTTAAGGGCTGGGGC	
9	F	ATTCCAGTGAAAGGGATTGAG	269
	R	ATCAGACCAGAGAAGTGACC	
10	F	AGGAGAGACCAGCGTGTACC	240
	R	CAGCAATAAACGGCGAGAAAC	
11	F	GGTCCATGGCAGCTTTGATG	217
	R	CTTATTCCCTGAGCCTCGAG	
12	F	AAAGGCTGCCCATCTGCTC	244
	R	CCTTGCTTTCTGTCTCCCAG	
13	F	GAGCTGGGCTGTGTCCTGCC	173
	R	AAGTTTGCTGCCTTTGATAT	
14	F	GCAGCCATAGGTCTTTGCTG	258
	R	ATGGAATCTCTTACACCTGG	
15	F	TCAAATTGTAAAGTCACCTAAAAGG	205
	R	AAGAGGGTTTTGTGCTGGAG	
16	F	CTTGATCAGTTCCTTCGTTGC	276
	R	GACTAAACTGTGGGGCTTTGC	

F, Forward; R, Reverse.

### Quantitative real-time polymerase chain reaction (qPCR)

qPCR was performed to detect exonic deletions/duplications and fine mapping of the breakpoints. Primers were designed with an amplicon size of less than 300 bp (Table 2). For fine mapping of the heterozygous deletion encompassing exon 2 identified in qPCR, additional primer pairs were designed and named In1 (located in intron 1), In2–1, In2–2, In2–3, In2–4, and In2–5 (located in intron 2; Table 2). The albumin (*ALB*) gene was used as the reference gene [19]. The qPCR reactions were performed in 10 µl volume containing 1×iQ^TM^ SYBR**^®^** Green Supermix (Bio-Rad Laboratories, Inc., Hercules, CA), primers at a final concentration of 0.25 μM each and genomic DNA at a concentration of 5 ng/μl. The qPCR reactions were run in the 7500 Real-time PCR system (Applied Biosystems, Foster City, CA). Cycling conditions were 95 °C for 10 min, and 40 cycles of 95 °C for 15 s and 60 °C for 1 min. A melting curve analysis of products was performed routinely following the amplification to test the specificity of the PCR products. Genomic DNA from a normal individual was used as a deletion-negative control (the calibrator sample) to normalization. Two independent experiments were performed. Quantitative PCR data were analyzed with the comparative threshold cycle method [20,21]. A relative quantity of about 1 in a normal sample, about 0.5 in a sample with a heterozygous *SAG* exon deletion, and about 1.5 in a sample with a heterozygous *SAG* exon duplication was expected.

**Table 2:** Primers for quantitative PCR analysis of *SAG*

**Name**	**F/R**	**Primer sequence**	**PCR size (bp)**
Exon1	F	CCTGGTTGGTGACAAATCACAAG	184
	R	CCGCTCACTCCAAGTCTCC	
Exon 2	F	ACACCCCAAGGTGGTAGAAGTT	123
	R	CACCACTCACCGATTTGTCC	
Exon5	F	CTCCCTTGCAGTGTATGTCAC	215
	R	AGTCACCCACCGTCAGGAGA	
In1	F	GAAAACAGTCTTTGCGAAGTGG	177
	R	ACACCTTGCCAGGGAGTCTA	
In2–1	F	TTCTTCTCCGTGCACCTACC	191
	R	AGCTATCCACAGAGGCTGGA	
In2–2	F	CCTCACAGGTGGGAAAAGAG	160
	R	GCTGGAGGATGAAGGTCAAAG	
In2–3	F	AACCACCAACTACCCTCGAC	171
	R	AAGGGGAGAACCAGTTAGGC	
In2–4	F	CCACAGAGAAGGAGTGAGCA	78
	R	CTGCCTTGCAGGAATGGTAAC	
In2–5	F	ACACCACCCAGGCTAGTGAG	149
	R	CTGTGCCCTCTAGGTTACTCG	

Quantitative PCR primers for other *SAG* exons are the same as the primers used for mutation screening except exon 1, exon 2, and exon 5 shown in this table. F, Forward; R, Reverse.

### Long-range polymerase chain reaction

To validate the heterozygous deletion detected with qPCR analysis, a long-range PCR approach was performed using the primers In1-F and In2–4-R (Table 2). The PCR conditions were 95 °C for 5 min, and 35 cycles of 94 °C for 30 s, 60 °C for 30 s, and 68 °C for 1 min, followed by the final extension at 68 °C for 5 min. The purified PCR products were directly sequenced.

## Results

### Clinical findings

**Figure 1:**
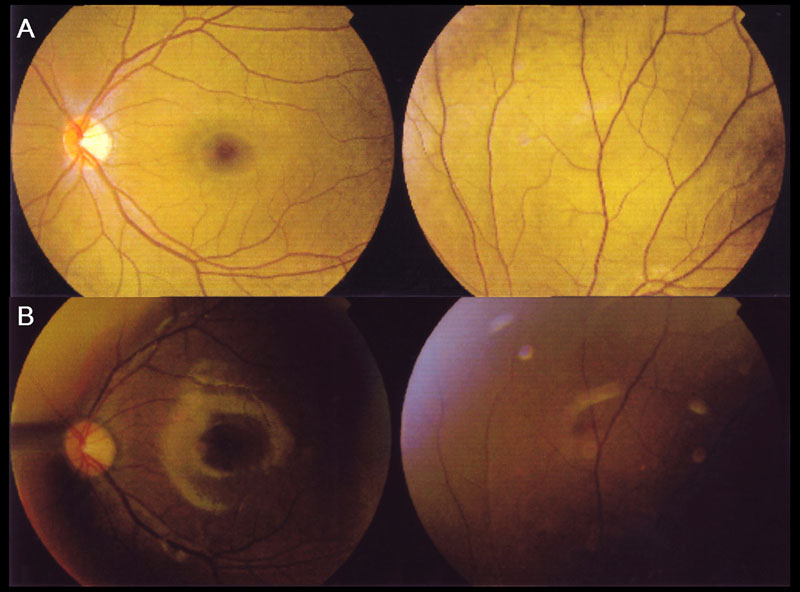
Fundus photographs before and after dark adaption in a Chinese patient with Oguchi’s disease. **A**: The fundus photographs revealed an abnormal golden-yellow reflex in the light-adapted state. **B**: This abnormal reflex disappeared after 4 h of dark-adaptation. The left eye is shown.

**Figure 2:**
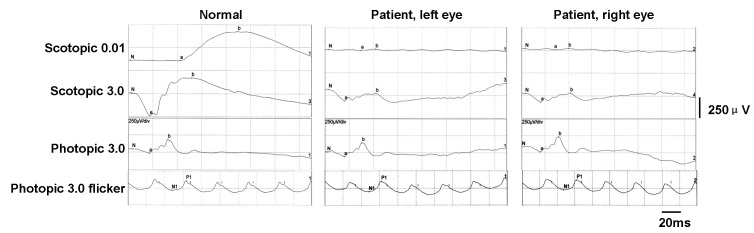
Full-field electroretinograms (ERGs) of a normal person and a patient with Oguchi’s disease. The rod b waves were nearly undetectable in scotopic 0.01 ERGs. The scotopic 3.0 ERGs show a significant reduction in a-wave amplitudes and a near total absence of b waves. The photopic 3.0 ERGs and photopic 3.0 flicker ERGs were almost normal.

**Figure 3:**
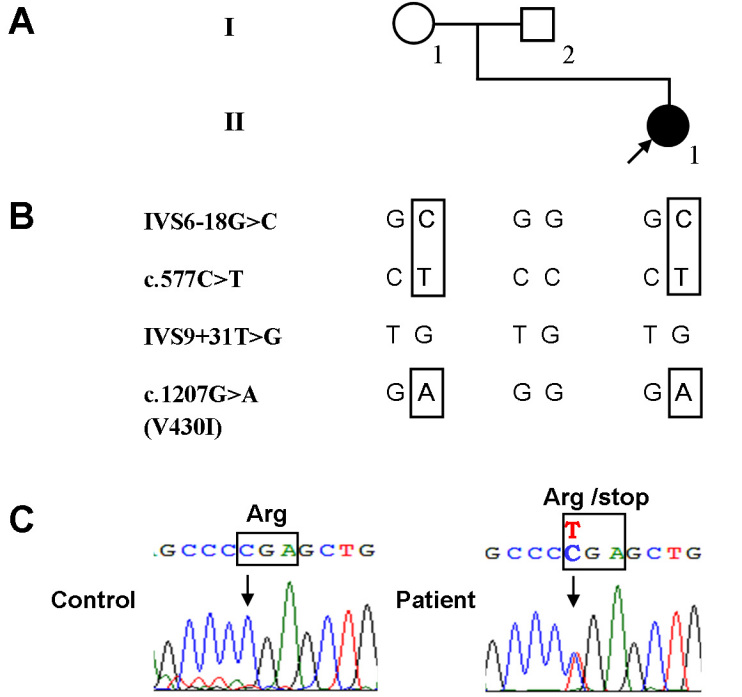
Mutation analysis of the *SAG* gene in a Chinese family with Oguchi’s disease. **A**: The family pedigree with the patient indicated with a filled symbol. **B**: Overview of the *SAG* nucleotide changes identified with mutation analysis in the family members. Nucleotide changes in the patient inherited from the mother are indicated in the box. **C**: Chromatograms showing the heterozygous *SAG* mutation c.577C>T (Arg193stop) in the patient. The affected genetic code is indicated in the box.

The proband was a 13-year-old girl who had night blindness since early childhood. Her BCVA was 1.5 in each eye. The refractive errors were −3.50 −0.50×180° in the right eye and −3.75 −0.50×180° in the left eye. Fundus examinations showed the characteristic golden-yellow discoloration in both eyes and the Mizuo-Nakamura phenomenon, in which the golden-yellow discoloration of the fundus (Figure 1A) disappeared after prolonged dark adaptation (Figure 1B). Neither vascular attenuation nor retinal degeneration was seen throughout the retina, and no maculopathy was observed. The visual fields of both eyes were within normal limits. The full-field scotopic 0.01 ERGs showed undetectable rod b waves in the patient. The scotopic 3.0 ERGs showed a “negative” configuration with a significantly reduced a wave and a nearly absent b wave in both eyes. The photopic 3.0 ERGs and photopic 3.0 flickers seemed almost normal (Figure 2). The girl was the first offspring of an unaffected non-consanguineous couple, and no family history was found in the family pedigree (Figure 3A).

### Molecular genetic findings

No mutations in the *GRK1* gene were found in the patient. Mutation screening of the *SAG* gene revealed a heterozygous nonsense mutation R193X (c.577C>T; Figure 3B,C), and three heterozygous single nucleotide polymorphisms (SNPs, recorded in the SNP database [build 130]) including IVS6–18G>C, IVS9+31T>G, and c.1207G>A (V430I; Figure 3B) in the patient. Among four nucleotide changes, the heterozygous nucleotide change IVS9+31T>G was seen in both parents while the other three changes were found only in the mother, suggesting those changes were inherited from the mother (Figure 3B). No putative pathogenic mutation was identified in the father.

**Figure 4:**
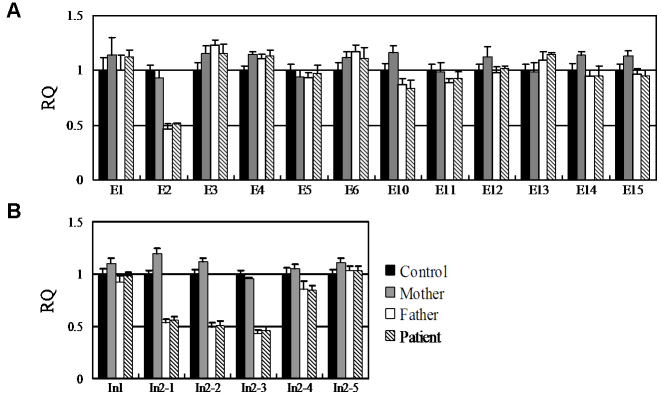
Relative quantity (RQ) value and standard deviation calculated from the data of quantitative real-time PCRs for detecting possible heterozygous deletions/duplications in the *SAG* exons in the patient and her father. An RQ value of about 1.0, 0.5, or 1.5 indicates the diploid genotype, heterozygous deletion, or heterozygous duplication, respectively. **A**: Identification of a heterozygous deletion encompassing the *SAG* exon 2 in the patient and her father. **B**: Validation and fine-mapping the deletion using the primers of In1, In2–1, In2–2, In2–3, In2–4, and In2–5 located in intron 1 and intron 2. The heterozygous deletion involved In2–1, In2–2, and In2–3. Data were based on two independent experiments. E, Exon; In, Intron. The error bar represents the standard deviation.

**Figure 5:**
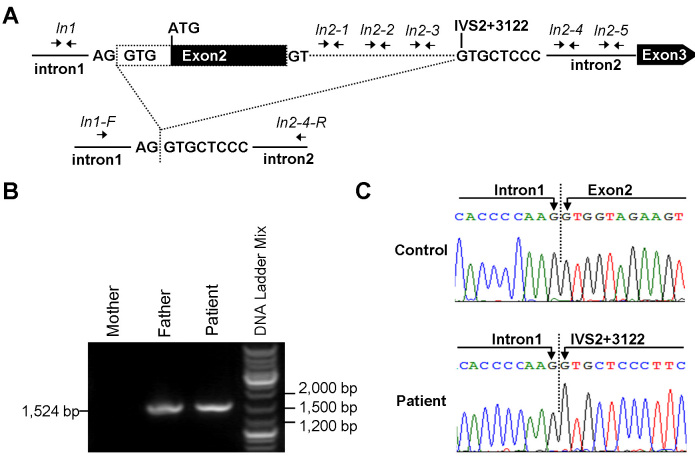
Identification of a novel heterozygous deletion containing the *SAG* exon 2 in the patient with Oguchi’s disease. **A**: Schematic representation of the identification of the intragenic deletion containing *SAG* exon 2 with the translation start site (ATG). Top: the location of primer pairs (In1, In2–1, In2–2, In2–3, In2–4, and In2–5) used in the quantitative PCR for fine mapping the breakpoints of the deletion in the region between intron 1 and exon 3. The deleted part of the *SAG* gene is shown with the dashed line. Bottom: the location of the primer pairs (In1-F and In2–4-R) used in long-range PCR. Boxes are exons with the coding region indicated in black and the 5′-untranslated region in white. The exons and introns are not drawn to scale. **B**: Gel analysis of the long-range PCR products. **C:** Chromatograms showing the breakpoints of the intragenic deletion.

The existence of four heterozygous nucleotide changes in the patient suggested that exon 7, exon 8, exon 9, and exon 16 were deletion-negative. qPCR analysis of the other 12 exons of the *SAG* gene was performed and revealed a heterozygous deletion of the *SAG* exon 2 in the patient and her unaffected father (Figure 4A). Fine mapping of the exon 2 deletion with qPCR analysis using the primers including In1, In2–1, In2–2, In2–3, In2–4, and In2–5 revealed the heterozygous deletion involving In2–1, In2–2, and In2–3 in the patient and her father (Figure 4B, Figure 5A).

Long-range PCR using the primers In1-F and In2–4-R amplified a shortened fragment of about 1,500 bp in the patient and her father (Figure 5B). Direct sequencing of the long-range PCR products revealed the breakpoints of the deletion, skipping a 3,224-bp fragment of the *SAG* gene. The 5′-end of the breakpoint was located in the intron 1/exon 2 boundary, and the 3′-end of the breakpoint was located in the intron 2, 3,122 bp downstream of exon 2 (IVS2+3122; Figure 5C). No shortened fragment was seen in 96 unrelated healthy controls.

## Discussion

Oguchi’s disease is a rare autosomal recessive inherited disease. Patients usually have typical clinical features including night blindness, typical fundus changes called the Mizuo-Nakamura phenomenon, and characteristic ERGs revealing reduced or no rod function with normal cone function [1-3]. The patient described here could be clinically diagnosed with Oguchi’s disease according to the typical ophthalmologic features revealed by clinical examinations.

Two causative genes, *SAG* [1] and *GRK1* [4], have been reported for Oguchi’s disease. To date, only four *SAG* mutations including 926delA, R193X, R175X, and R292X have been reported in 16 Japanese families [1,8-12,14,16], one Indian family [13], and one South Asian family [15] with Oguchi’s disease. The most recent report of Oguchi’s disease was identified in a Japanese patient with only one allele of the frameshift 926delA mutation [16], suggesting that other types of mutation, undetectable by mutation screening of the coding regions of the *SAG* gene, may exist in the other allele of the *SAG* gene in the patient.

In our study, only one heterozygous nonsense R193X mutation was identified in the patient and her mother by mutation screening of the coding regions of the *SAG* gene. The homozygous nonsense R193X mutation has been reported in only one Indian family with Oguchi’s disease. qPCR analysis followed by long-range PCR revealed a novel heterozygous deletion of 3,224 bp, encompassing exon 2 and partial intron 2, in the patient and her father. To our knowledge, this is the first report of a novel heterozygous deletion in the *SAG* gene identified using qPCR and the first case of Oguchi’s disease found in a Chinese family.

**Figure 6:**
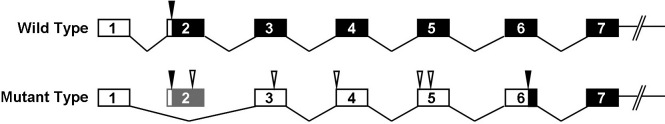
Schematic representation of the mature mRNA of wild type and mutant type with exon 2 and the natural AUG codon eliminated. The in-frame coding regions are indicated in black box. The 5′ untranslated regions and out-frame coding regions are in clear box. The locations of in-frame AUG codons and out-of-frame AUG codons are indicated with triangles in black and in white, respectively. The numbers in the boxes depict the exons.

This deletion was predicted to eliminate the entire exon 2 of 103 nucleotides (nt) with the AUG initiation site in the mature mRNA of *SAG* and cause the use of downstream alternative AUG codons (Figure 6). The eukaryotic ribosomes are loaded on the 5′-cap of the mutant mRNA, scan for the translation initiation signal (TIS), and initiate the first AUG codon they encounter [22]. The first downstream AUG codon out of frame located in the exon 3 is recognized by the ribosome, initiating the translation of its open reading frame (ORF). A short peptide of 19 amino acids is produced, which is expected to stall ribosomes to its stop codon [23,24]. In addition, the premature termination codon leads to the rapid mRNA degradation by the pathway of the nonsense-mediated mRNA decay (NMD) [25]. If possible, the mRNAs if not all degraded may remain connected to the un-disassociated ribosomes stalled at the stop codon of the ORF, and thus, these ribosomes may resume scanning and reinitiate the next downstream TIS [26,27]. In this case, there would be three additional downstream out-of-frame AUG codons to be scanned by the ribosomes in the same way. And if the fifth in-frame AUG codon has a chance to be initiated, a predicted protein lacking 134 amino acids in the NH_2_ terminus would be expressed in a dramatically reduced level. Even if the expression level is high enough, the predicted truncated protein could be non-functional with impaired conformation of the NH_2_-terminal half of the SAG protein and disrupted interaction between the N domain and the C domain [28-31].

Interestingly, no missense mutations have been reported so far. All the previously reported mutations, nonsense or frameshift, were predicted to produce truncated proteins lacking the COOH terminus if the transcripts of the gene were not all degraded by NMD [1,8-16]. Even if the expression level is high enough, the predicted truncated proteins could be non-functional [1,8-16]. Taken together with the novel deletion reported in this study, these results led us to suggest that the predicted aberrant *SAG* gene products with a loss of either the COOH-terminus or the NH_2_-terminus caused by nonsense mutations, frameshift mutations, or large fragment deletions eliminating natural AUG codon, having great potential to impair or abolish the function of the SAG protein and thus cause its transcripts to be exposed to the NMD [25], will cause Oguchi’s disease. Missense mutations that are not the target of NMD may remain some basic function of the SAG protein and therefore cause mild Oguchi’s disease without significant night blindness. However, more reports of Oguchi’s disease may help to elucidate the underlying mechanism.

Recently, two recorded copy number variants in the Database of Genomic Variants (DGV), Gain_79017 [32] and Loss_53177 [33], have been identified in normal individuals with incidence of 2 in 90 (>2.2%) and 2 in 1,854 (1,064 plus 790; approximately 1‰), respectively. Those two variants could be potential hot-spot rearrangement sites for Oguchi’s disease. The duplication of the exon 3 (Gain_79017) is predicted to be pathogenic by inserting 61 nucleotides in the coding region of the mRNA transcripts, causing a frameshift effect and triggering the NMD. The loss of the exon 11 to 16 (Loss_53177) is also predicted to be pathogenic by lacking the function of the C-terminus. Therefore, the incidence of Oguchi’s disease caused by the *SAG* gene mutations could be higher than expected. The relative low number of reports on *SAG* gene mutations could partly be explained by the putative pathogenic *SAG* deletions/duplications that escape the most common mutation screening methods for detecting point or minute mutations. The identification of the first deletion in the *SAG* gene described in this study will suggest the idea that the search for possible rearrangements in the *SAG* gene should be routinely performed in all patients with Oguchi’s disease if there is a negative result of the mutation screening of the *SAG* gene in one or two alleles.

In summary, the compound heterozygosity for a heterozygous nonsense mutation and a heterozygous deletion of 3,224 bp encompassing exon 2 in the *SAG* gene would putatively explain Oguchi’s disease in a Chinese family. Here, we emphasize that qPCR analysis should be performed if the mutation screening of the *SAG* gene fails to detect the aberrant variants in patients with Oguchi’s disease. The novel heterozygous deletion in the *SAG* gene identified with the qPCR method in this study will expand the spectrum of *SAG* mutations associated with Oguchi’s disease, and this will help to elucidate further the role of this gene in the etiology of Oguchi’s disease.
